# Oxytocin and Food Intake Control: Neural, Behavioral, and Signaling Mechanisms

**DOI:** 10.3390/ijms221910859

**Published:** 2021-10-08

**Authors:** Clarissa M. Liu, Mai O. Spaulding, Jessica J. Rea, Emily E. Noble, Scott E. Kanoski

**Affiliations:** 1 Neuroscience Graduate Program, University of Southern California, Los Angeles, CA 90089, USA; clarissa.ming.liu@gmail.com (C.M.L.); jjrea@usc.edu (J.J.R.); 2 Human and Evolutionary Biology Section, Department of Biological Sciences, University of Southern California, Los Angeles, CA 90089, USA; 3 Department of Nutritional Sciences, University of Georgia, Athens, GA 30606, USA; mai.spaulding@uga.edu

**Keywords:** oxytocin, obesity, reward, satiation, meal size, energy balance, hindbrain, sugar, feeding

## Abstract

The neuropeptide oxytocin is produced in the paraventricular hypothalamic nucleus and the supraoptic nucleus of the hypothalamus. In addition to its extensively studied influence on social behavior and reproductive function, central oxytocin signaling potently reduces food intake in both humans and animal models and has potential therapeutic use for obesity treatment. In this review, we highlight rodent model research that illuminates various neural, behavioral, and signaling mechanisms through which oxytocin’s anorexigenic effects occur. The research supports a framework through which oxytocin reduces food intake via amplification of within-meal physiological satiation signals rather than by altering between-meal interoceptive hunger and satiety states. We also emphasize the distributed neural sites of action for oxytocin’s effects on food intake and review evidence supporting the notion that central oxytocin is communicated throughout the brain, at least in part, through humoral-like volume transmission. Finally, we highlight mechanisms through which oxytocin interacts with various energy balance-associated neuropeptide and endocrine systems (e.g., agouti-related peptide, melanin-concentrating hormone, leptin), as well as the behavioral mechanisms through which oxytocin inhibits food intake, including effects on nutrient-specific ingestion, meal size control, food reward-motivated responses, and competing motivations.

## 1. Introduction

Oxytocin is a nine-amino-acid neuropeptide produced in the paraventricular hypothalamic nucleus (PVH) and supraoptic nucleus of the hypothalamus (SON) that acts on the G-protein coupled oxytocin receptors to impact several behaviors, including social behavior, reproduction, and lactation [1,2,3]. It is now well established that central oxytocin also modulates food intake. For example, pharmacological injection of oxytocin into the brain reduces food intake whereas administration of an oxytocin receptor antagonist has the opposite effect in rodents [4,5,6,7,8,9,10,11,12,13]. Additionally, oxytocin receptor null mice demonstrate increased food intake during the nocturnal cycle [14], and virally-mediated knockdown of PVH oxytocin mRNA expression increases both low-fat and high fat diet intake [15]. Oxytocin is regulated by single-minded homologue 1 (SIM 1), a transcription factor involved in the development of the PVH [16]. While mice with a homozygous null allele of SIM 1 die perinatally, heterozygous mice develop early-onset obesity with increased hyperinsulinemia and hyperleptinemia [17]. However, chronic pharmacological treatment of oxytocin results in the reversal of hyperphagia and obesity in SIM 1 haploinsufficient mice [18], thus further supporting oxytocin’s role in regulating energy balance.

Collective evidence suggests that oxytocinergic regulation of eating behavior is important for satiation and meal size control rather than modulating interoceptive hunger and satiety state. For example, peripheral oxytocin administration does not affect perceived levels of hunger or satiety in a food deprivation discrimination procedure in rats, despite reducing food intake in both food-restricted and non-restricted states [19]. Further, oxytocin’s pharmacological effects involve reduction in meal size (see Section 2.1 and Section 5.2 for additional discussions), oxytocin neurons are activated coinciding with meal cessation, and oxytocin gene expression is elevated upon re-feeding [18,20]. Due to its potent effects on reducing meal size and body weight in both rodent and human studies [4,21,22,23], oxytocin is currently under clinical investigation as a therapeutic for obesity treatment [24,25,26,27,28,29,30,31].

Several questions remain unanswered regarding the mechanisms and sites of action through which oxytocin modulates energy balance. In this review we focus on the current state of the literature pertaining to central oxytocinergic signaling and effects on eating. We discuss known neural sites of action, oxytocin’s interactions with various other feeding-related peptides in the control of food intake, and volume transmission as a possible neural signaling pathway through which oxytocin influences eating behavior. We further review evidence related to behavioral mechanisms through which oxytocin inhibits food intake, including effects on satiation control, brain reward signaling pathways, and competing motivations.

## 2. Neural Sites of Action for Oxytocin’s Effects on Food Intake

Oxytocin neurons in the PVH and SON project widely throughout the brain. Immunohistochemistry-based tracing from these neurons has identified projections to various food intake-relevant nuclei, including (but not limited to) the medial preoptic area (MPOA), bed nucleus of the stria terminalis (BST), septal nuclei, prefrontal cortex (PFC), nucleus accumbens (ACB), central (CEA) and medial amygdala (MEA), basolateral amygdala (BLA), hippocampus, ventral tegmental area (VTA), nucleus tractus solitarius (NTS), and the dorsal nucleus of the vagus nerve (DMV) [1,32,33,34]. While these projections highlight a mechanism for oxytocin-mediated anorexigenic effects via synaptic axonal release at distal targets, additional signaling modalities exists wherein oxytocin release can also occur following somatic or dendritic release, and possibly via release into the cerebrospinal fluid (see Section 3 for additional discussion). In support of these alternate pathways, the oxytocin receptor is expressed in additional eating-relevant brain areas that receive minimal or no oxytocinergic projections, such as the ventromedial nucleus of the hypothalamus (VMH) [11,35]. Therefore, below we review evidence for site-specific effects of oxytocin on eating behavior in sites of action that receive direct oxytocin neuron innervation as well as regions containing the oxytocin receptor yet lack dense innervation from oxytocin neurons.

### 2.1. Caudal Brainstem

Receiving direct oxytocinergic input, the caudal brainstem is a critical region for oxytocin’s anorexigenic effects. Within this region, NTS neurons are an essential hub for energy balance control and integrate vagally-mediated gastrointestinal (GI) satiation signals, hormonal and nutrient signals in the blood, with descending input from the forebrain [36]. Recent evidence from rodent models demonstrates that oxytocin receptor (OT-R) signaling in the medial NTS (mNTS) reduces chow intake in a dose-dependent manner, and an interaction between mNTS OT-R signaling and meal-related gastrointestinal (GI) nutrient processing (e.g., induced via preload ingestion) contributes to further food intake reduction [12,37,38]. Consistent with these findings, fourth ventricular (restricted to hindbrain) oxytocin receptor antagonism reduces the anorexigenic effect of GI-derived satiation signals, such as leptin and cholecystokinin (CCK) [37,39], and modulates visceral vagal afferent-evoked neural activity [40]. These outcomes are likely mediated by a direct synaptic pathway from oxytocin neurons, as parvocellular PVH oxytocin fibers innervate the NTS [41]. Oxytocin may also engage the caudal brainstem indirectly via action on OT-Rs expressed in the vagal sensory neurons (nodose ganglia) [42], as the increased c-Fos expression in the NTS and the reduced food intake following peripheral oxytocin administration are each blocked in vagotomized animals [43].

Not surprisingly, oxytocin’s action in the hindbrain reduces food intake primarily via a reduction in meal size. For example, mNTS OT-R knockdown (KD) in rats yields larger spontaneous meal size consumption with a compensatory reduction in meal frequency such that cumulative caloric intake is not affected [38]. Considering GI signals contribute to meal size control [44,45], these results support the hypothesis that NTS OT-R signaling augments the efficacy of GI-derived satiation signals. In addition, consistent with this model, NTS oxytocinergic projections are upregulated following primary adrenalectomy, which is also associated with reduced meal size [46]. In addition to oxytocin acting in the NTS to amplify vagally-mediated satiation signals, Wald and colleagues revealed that NTS oxytocin signaling reduces conditioned motivated behaviors for palatable food. Specifically, oxytocin administration to the NTS reduces motivation to work for sucrose in a progressive ratio schedule of operant reinforcement, and reduces reinstatement of palatable food-seeking behavior [47]. Altogether, these data indicate that NTS OT-R signaling is important for amplification of GI signals in the control of both satiation and food-motivated behavior.

### 2.2. Hypothalamus (Arcuate Nucleus and Ventromedial Nucleus of the Hypothalamus)

In addition to the caudal brainstem, oxytocin acts within several hypothalamic nuclei to regulate energy balance. The arcuate nucleus of the hypothalamus (ARH), perhaps the most widely studied brain nucleus for the control of eating behavior, sends dense projections to PVH oxytocin neurons [48,49]. Within the ARH there are two opposing neuronal subtypes that potently regulate food intake and energy balance: (1) proopiomelanocortin/cocaine- and amphetamine-regulated transcript (POMC/CART) neurons which inhibit food intake, and (2) the neuropeptide-Y/agouti-related peptide (NPY/AgRP) neurons which stimulate food intake. POMC is a precursor protein for α-melanocyte stimulating hormone (α-MSH), which activates PVH magnocellular oxytocin neurons [48] and increases secretion of oxytocin [50]. Central (intracerebroventricular; ICV) or PVH administration of α-MSH potently inhibits food intake [51] and induces c-Fos expression in oxytocin neurons [52]. Interestingly, while ARH-derived neuronal peptides can activate oxytocin neuronal c-Fos and/or increase oxytocin secretion, oxytocin injection into the ARH also reduces food intake [53]. The critical interaction between ARH-derived α-MSH and central oxytocin signaling is further supported by results showing that the anorexigenic effects of central administration of α-MSH are attenuated by pretreatment with an oxytocin receptor antagonist [54]. On the other hand, NPY/AgRP neurons in the ARH inhibit oxytocin neurons, thereby contributing to increased food intake [55].

In contrast to the ARH, the VMH is not apparently innervated by oxytocinergic terminals, yet this region does contain the oxytocin receptor [56]. Moreover, oxytocin increases the firing activity of ventrolateral VMH neurons [57] and oxytocin reduces food intake when administered in the VMH [11,58]. Interestingly, VMH oxytocin administration does not affect intake of sweet and palatable saccharin and sucrose solutions, which is consistent with findings that c-Fos activated sites following VMH oxytocin injection are primarily in hypothalamic regions (e.g., ARH, PVH) but not sites linked with reward processing, such as the ACB and VTA [58] (see Section 2.3 below for discussion on oxytocin’s direct action in the ACB and VTA). The mechanisms by which oxytocin reaches the VMH given low levels of innervation remain to be identified.

In addition to reducing food intake, VMH oxytocin administration also increases short-term energy expenditure and spontaneous physical activity [11], an effect that is more pronounced in females during the proestrus stage of the estrus cycle due to estrogen mediated elevation of the OT-R [59]. Indeed, there is an abundance of evidence indicating that OT-R expression in the VMH is modulated by reproductive hormones, such as testosterone, estrogen, and dihydrotestosterone [60,61,62]. For example, estrogen pretreatment in ovariectomized (OVX) female rats augments VMH oxytocin-induced running activity [59]. It has been proposed that oxytocin-induced changes in energy expenditure and locomotion are based on the role of VMH oxytocin in reproductive behavior, and thus it may be that a reduced eating drive functions to offset a competing motivation [59]; however, this idea remains to be experimentally tested.

### 2.3. Ventral Tegmental Area and Nucleus Accumbens

In addition to acting in brain regions, such as the caudal brainstem and hypothalamus that regulate “homeostatic” or energy need-based aspects of food intake, there is growing evidence that oxytocin acts within the brain reward circuitry to suppress consumption of and motivated responding for palatable food. In both rodents and humans, oxytocin administration preferentially reduces intake for sweet-tasting carbohydrate-based food [63]. Moreover, oxytocin knockout (KO) mice demonstrate enhanced intake of palatable sucrose solutions, as well as increased operant responding for sucrose in a progressive ratio paradigm [64]. In the mesolimbic pathway, the VTA sends dopaminergic projections to the ACB to affect motivated aspects of eating behavior. Both the VTA and ACB receive oxytocin neuron projections and densely express oxytocin receptors [65,66]. Further, numerous studies have shown that central oxytocin preferentially reduces intake of and motivated responding for sweet-tasting palatable foods, in part, through actions on the VTA and ACB [7,10,64,67,68]. For example, recent evidence demonstrates that direct administration of an OT-R antagonist in the VTA significantly increases sucrose intake [10], and VTA OT-R agonism reduces sucrose motivation and chocolate pellet seeking [47]. A potential molecular mechanism through which oxytocin exerts these effects may be through modulation of dopaminergic signaling. Indeed, about 10% of OT-R-expressing VTA neurons are dopaminergic and these neurons project to the ACB [65]. Recent evidence from our group revealed that ICV oxytocin administration suppresses phasic dopamine neuron activity in the VTA in response to cues associated with sucrose [69]. The modulation of VTA excitatory transmission is pathway specific and may be limited to cells that express the cannabinoid receptor 1, as OT-R signaling stimulates endocannabinoid release from dopamine neurons, which acts on excitatory glutamatergic inputs to VTA neurons to suppress glutamatergic transmission [66,70]. While the impact of this cannabinoid-dependent signaling on food intake control remains to be determined, this molecular mechanism is analogous to the way in which insulin induces synaptic long-term depression of mouse VTA DA neurons and reduces food anticipatory behavior [71]. Another potential mechanism by which oxytocin modulates dopamine activity is through either direct activation or indirect inhibition of VTA and substantia nigra pars compacta (SNc) DA neurons [70,72].

While OTR-expressing VTA neurons directly project to the ACB [65], oxytocin also directly acts in the ACB core, but not the ACB shell, to reduce food restriction-induced chow intake and consumption of palatable sucrose and saccharin solutions in nondeprived animals [7]. Furthermore, the anorexigenic dose of oxytocin in the ACB core does not induce conditioned flavor avoidance [7], suggesting that oxytocin does not produce malaise when injected into this region. Interestingly, ACB oxytocin signaling also reduces drug reinforcement, as evidenced by reduced methamphetamine seeking and motivated responding [73]. In contrast to a reduced motive for food and drug reward, a recent study revealed that oxytocin acts in coordination with serotonin to facilitate social reward via modulation of the ACB core synaptic plasticity [74,75]. Interestingly, the anorexigenic effect of ACB oxytocin is reduced in a social context [7]. Together, these data suggest that oxytocin acts in the VTA and ACB to modulate dopamine neuron signaling to enhance social reward but reduces the reinforcing properties of palatable foods and drugs of abuse, potentially by reducing phasic dopamine neuron responses to food conditioned cues. The precise molecular mechanisms through which these competing outcomes interact require future study.

### 2.4. Amygdala and Hippocampus

The amygdala is critical for resolving eating versus threat avoidance competition and for integrating learned food cues [76]. Magnocellular oxytocin neurons project to the amygdala, and axonal release of oxytocin in the CEA can attenuate conditioned fear responses [77]. While it is well established that OT-R signaling in the amygdala plays an important role in stress responses [77,78] and social behavior [79,80], recent evidence demonstrates that amygdala OT signaling produces a moderate yet significant reduction in food intake. For example, oxytocin administration in the BLA or CEA reduces standard chow intake in rats re-feeding after food restriction, and pretreatment with an OT-R antagonist attenuates these anorexigenic effects [8]. While high doses of oxytocin in the amygdala can produce conditioned taste aversion [81], oxytocin at these smaller anorexigenic doses does not, suggesting that the anorexigenic effects of oxytocin in this region are not secondary to malaise [8]. However, while both CEA and BLA oxytocin administration reduces chow intake after a fast, only BLA oxytocin suppresses consumption of sucrose and saccharin solutions [8]. Given the amygdala’s role in processing food-associated cues, future studies examining the role of amygdala oxytocin signaling in motivated responding for palatable food and cue-induced food seeking is warranted.

The hippocampus also receives direct input from oxytocin neurons, albeit minimal, and expresses the oxytocin receptor in both the dorsal and ventral subregions of the rat, particularly in field CA1 [82,83,84]. Considerable research has focused on the effects of hippocampal oxytocin signaling on social behavior (e.g., social recognition) [85,86]. However, to our knowledge the role of hippocampal oxytocin receptor signaling on food intake and food-motivated behavior has not been investigated. Such analyses are warranted as the hippocampus has recently emerged as an important brain structure in regulating food intake. For example, in addition to oxytocin, hippocampal neurons express receptors to various endocrine and neuropeptide signals that regulate food intake, including CCK, melanin-concentrating hormone (MCH), insulin, leptin, ghrelin, glucagon-like peptide-1 [87]. It has been proposed that the hippocampus, particularly the ventral subregion, integrates external visuospatial cues, internal energy status-related contextual information, and learned experiences to bidirectionally control eating and food-motivated behavior [87,88]. Preliminary data from our lab reveal that doses of oxytocin that are subthreshold for effects in the cerebral ventricles yield a modest, yet significant reduction in nocturnal chow intake when administered to either the dorsal or ventral hippocampal subregion in rats. Based on these preliminary findings and the growing number of publications identifying a role for memory processing in food intake control [89], we highlight this region as an understudied brain region with regards to oxytocin’s anorexigenic effects.

## 3. Volume Transmission of Oxytocin

In contrast to wiring transmission, where fast intercellular communication occurs between synapses and gap junctions, volume transmission is a slower modulatory form of intercellular communication, in which cell transmission of signaling molecules occurs via the interstitial and/or the cerebrospinal fluid (CSF) of the brain [90,91]. Oxytocin has been postulated to be transmitted via volume transmission based on the fact that the majority of oxytocin in magnocellular neurons is stored in dendrites and not axon terminals, and the location of these dendrites within the PVH penetrate the ventricular space and have been shown to transmit oxytocin into the third ventricle [92,93]. Additional evidence corroborating volume transmission as an important mode of oxytocinergic communication is the presence of brain regions where an oxytocin receptor-terminal mismatch exists. For example, a high density of oxytocin receptor is found in the hippocampus and VMH, but there are minimal oxytocin projections to either region [94]. Together these findings suggest that oxytocin could potentially circulate to distal brain regions in CSF, and/or that CSF- or parenchymal-released oxytocin could potentially have a modulatory impact by transmission through the interstitial space. Indeed, recent evidence shows that oxytocin neurons of the SON secrete oxytocin from dendrites [95], and that dendritic secretion of oxytocin from SON neurons in the medial amygdala is essential for social recognition memory [96]. Furthermore, oxytocin is released from axonal varicosities originating from the PVH where they diffuse through the extracellular space to activate gastrin-releasing peptide neurons of the lumbar sacral spinal cord to modulate male sexual arousal [97]. Together these findings demonstrate that volume transmission of oxytocin is a relevant mode of communication for modulating physiological processes.

While the above evidence shows that oxytocin is transmitted via volume transmission, the impact of this signaling modality to the central regulation of food intake control remains to be determined. We recently showed that another hypothalamic neuropeptide known to affect food intake, MCH, is transmitted via the CSF to modulate eating behavior [98], and thus it is possible that other neuropeptides also utilize the CSF for transmitting appetite-relevant signals. Indeed, oxytocin has been detected in the CSF of rodents and humans, with state dependent fluctuations in concentration and a long half-life in the CSF compared to that in blood (~28 min vs. ~2 min) [92,99,100,101]. Future research is required to understand whether oxytocin release in CSF is a signaling pathway relevant to the anorexigenic effects of this peptide system.

## 4. Interactions with Energy Balance-Associated Peptides

A burgeoning body of research demonstrates that oxytocin interacts with various endocrine and neuropeptide systems to regulate food intake, some of which were briefly touched upon above. Indeed, the literature suggests that “hunger” signals initially silence oxytocin responses [55], and “satiation” signals enhance oxytocin activity to signal meal termination [37,102]. Below we review the current literature exploring the role of oxytocin’s interaction with neural and gut-derived peptides in the control of food intake.

### 4.1. AgRP (NPY)/POMC (CART)

As described above, AgRP and POMC neurons in the ARH potently regulate food intake. AgRP neurons integrate peripheral signals to stimulate feeding and directly project to the PVH where they inhibit oxytocin neurons [55,103,104]. This neural pathway may be a rapid and short-acting response to initiate food intake at mealtime. Another putative consequence of reduced oxytocin neuron activity due to increased AgRP signaling is attenuated conditioned taste aversion (CTA) learning. For example, AgRP injection into the lateral ventricle impairs acquisition of CTA, with corresponding reductions in the percentage of c-Fos positive oxytocin neurons induced by lithium chloride (an agent that produces malaise and a robust CTA) [105,106]. Thus, these data suggest that the AgRP system engages the oxytocin neurons in an inhibitory manner, impacting both rapid eating responses and learned flavor-malaise pairing.

Not only do ARH AgRP neurons modulate oxytocinergic signaling, but ARH oxytocin signaling counteracts the behavioral consequences of AgRP signaling, as evident from findings that oxytocin reduces food intake when injected into the ARH [53]. Oxytocin receptor containing neurons in the ARH were identified as glutamatergic neurons that rapidly induce satiation and project to melanocortin-4 receptor (MC4R)-expressing neurons in the PVH [107]. Thus, collectively these findings demonstrate that ARH AgRP and oxytocin signaling have opposing effects on food intake via multiple neural pathways.

### 4.2. MCH

MCH is a hypothalamic neuropeptide that increases food intake and promotes weight gain (for review see [108]). Additionally, MCH plays a role in higher-order and learned aspects of eating and food-motivated processes by promoting behaviors such as food impulsivity and cue-potentiated eating [109,110]. Oxytocin neurons express the MCH 1-receptor (MCH1R) [111] and MCH modulation of oxytocinergic signaling has been shown to affect diverse range of physiological and behavioral functions, including lactation [112,113] and mood regulation [114]. Further, MCH signaling facilitates oxytocin-induced reduction in repetitive, stereotypic behaviors and social recognition memory [115,116].

In addition to MCH regulation of oxytocin signaling, there is evidence for oxytocinergic regulation of MCH neuronal signaling. For example, oxytocin neurons directly project to MCH neurons and ~60% of MCH neurons express oxytocin receptors [116]. Moreover, oxytocin excites GABA neurons that contain MCH [117], and targeted oxytocin receptor deletion from MCH neurons in mice leads to a decrease in primary inputs from the PVH, LHA, ACB, and VTA, suggesting that oxytocin plays an important role in plasticity and circuit formation with MCH neurons [115]. To our knowledge, however, the interaction between MCH and oxytocin in the control of food intake has not been explored. Given that both of these hypothalamic peptides systems have potent and opposing actions on food intake understanding their potential interaction in the control of energy balance represents an important area for future studies.

### 4.3. Leptin

Leptin, secreted from adipose tissue, crosses the blood–brain barrier, and acts in the brain to suppress food intake [118]. Systemic administration of leptin increases the electrical activity of oxytocin neurons [102], and ICV administration of leptin activates STAT3 phosphorylation (an intercellular marker for leptin receptor signaling) in a subpopulation of oxytocin neurons in the PVH that innervate the NTS [39,119]. These data support the hypothesis that leptin acts via a downstream oxytocinergic pathway to reduce food intake. Further supporting this concept, ventricular administration of an oxytocin receptor antagonist attenuates the effect of leptin on reducing food intake [39]. One study demonstrated that oxytocin administration alleviates acute but not chronic leptin resistance in diet-induced obese mice [120]. On the other hand, another study revealed that chronic treatment of oxytocin (via osmotic minipumps implanted subcutaneously) in leptin-resistant Zucker Fatty rats decreases food intake and body weight [121]. The discrepancies in these studies could be due to species differences and/or routes of drug administration. Overall, however, it appears that leptin acts directly on oxytocin neurons, and that oxytocin acts as a sensitizer to leptin effects on food intake. Whether pharmacological oxytocin can offset the leptin resistance associated with obesity requires further investigation.

### 4.4. CCK

CCK is primarily synthesized and released by enteroendocrine cells in the jejunum and duodenum soon after food reaches the small intestine [122,123,124]. Peripheral administration of CCK inhibits food intake via reductions in meal size, a process that is vagally-mediated [44,125]. Recent studies have shown that oxytocin and CCK interact within the NTS to reduce food intake. Specifically, rats that receive an anorexigenic dose of peripheral CCK show c-Fos activation in NTS regions that receive dense oxytocin axon innervation [37]. Moreover, the intake-inhibitory effects of peripheral CCK are attenuated by hindbrain OT-R antagonism [37]. Correspondingly, CCK enhances oxytocin functionality. For example, CCK stimulates oxytocin release [126,127,128,129], and peripherally administered CCK-8 and secretin activates oxytocin neurons to reduce both food and water consumption [130] via a possible noradrenergic mechanism [131]. Interestingly, a taste stimulus, specifically sucrose, previously paired with central administration of CCK induces oxytocin release [132], suggesting that central CCK signaling promotes the conditioned release of oxytocin. Together these data demonstrate that oxytocin and CCK systems act in a coordinated fashion to increase vagally-mediated satiation signaling.

### 4.5. GLP-1

Like CCK, glucagon-like peptide 1 (GLP-1) is an intestinally derived peptide that enhances vagally-mediated satiation signaling. GLP-1 is also synthesized from neurons in the caudal brainstem, and recent data reveal that the peripheral and central GLP-1 systems reduce food intake via distinct signaling pathways [133]. Oxytocin-positive terminals are in close apposition with brainstem GLP-1 positive perikaryal, and central infusion of oxytocin induces c-Fos expression in GLP-1-producing neurons [134]. Additional evidence supports the notion that central GLP-1 acts downstream of central oxytocin to reduce food intake. More specifically, while central infusion of a GLP-1 receptor antagonist followed by anorexigenic dose of oxytocin eliminated the anorexigenic effect of oxytocin, central infusion of an oxytocin receptor antagonist followed by synthetic GLP-1 ligand does not affect GLP-1-induced anorexia [134]. Together these results suggest that oxytocin modulates hindbrain GLP-1 neuron signaling to inhibit food intake and that GLP-1-mediated activation of PVH neurons likely acts through non-oxytocinergic pathways to control food intake. Additional studies are required to investigate possible interactions between the peripheral GLP-1 system and oxytocin-mediated food intake reductions, particularly given there several long-acting GLP-1 analogs are FDA-approved for obesity and diabetes treatment.

## 5. Behavioral Mechanisms Mediating Central Oxytocin’s Anorexigenic Effects

### 5.1. Nutrient-Specific Effects on Intake

Oxytocin release is triggered in response to physiological cues such as gastric distension and increased plasma osmolality [135], suggesting that oxytocin inhibitory effects on food intake can be independent of calorie, nutrient, and/or flavor components of the food. However, more recently it has been suggested that oxytocin may modulate intake in a macronutrient-dependent manner [63]. Specifically, it has been suggested that oxytocin may preferentially reduce the intake of palatable, sweet foods [7,8,10,64,68,136]. Studies using injections of the oxytocin receptor antagonist, L-368,899, which crosses the blood–brain barrier, demonstrate that OT-R blockade preferentially increases intake of carbohydrates, and OT KO mice demonstrate increased preference for carbohydrates but not fat [10,137,138]. Similarly, oxytocin KO mice exhibit heightened preference for sucrose and palatable isocaloric carbohydrate solutions independent of sweetness intensity, as well as for non-caloric carbohydrate sweetener (saccharin) [64,68]. Relative to wild type controls, these mice also overconsume sucrose and saccharin solutions when presented ad libitum as a two-bottle choice with water [136]. Conversely, oxytocin KO mice do not overconsume palatable intralipid solution, suggesting that lipid intake may not engage the oxytocin signaling pathway [64,68]. A possible mechanism for increased intake of sweet foods in oxytocin KO mice may occur through altered taste perception as OT-R is expressed in taste buds [139]. In support of this idea, intraperitoneal (IP) injection of oxytocin suppresses licking for sucrose, but not NaCl, quinine, or citric acid, and this response correlates with sucrose concentration [140].

In the brain, magnocellular oxytocin neurons in the PVH are activated following consumption of 10% sucrose but not following consumption of 4.1% intralipid with equivalent consumption of tastants [138]. Similarly, oxytocin gene expression is elevated in the hypothalamus by consumption of carbohydrates but not intralipid [138]. Further, rats that were given sweetened condensed milk showed increased activation of oxytocin neurons in both the SON and PVH whereas a cream gavage (high in fat without added sugar) does not have a compelling effect on oxytocin neuronal activity [141]. Other studies have shown that consumption of sucrose leads to elevated oxytocin mRNA, and administration of an OT-R antagonist consistently produces elevation of carbohydrate intake in choice and no-choice food intake paradigms [137]. Taken together, these data suggest that palatable carbohydrates activate oxytocin neurons in the brain, whereas foods high in lipids have a substantially smaller effect.

Oxytocin’s macronutrient specific effects may depend on the site of action. For example, injection in the BLA has been shown to reduce sucrose and 0.1% saccharin intake [8]. Saccharin is an artificial sweetener devoid of calories, and thus reduction in saccharin intake suggests that oxytocin in the BLA reduces intake independent of calories and potentially driven by hedonic gustatory processing. Additionally, reduction of sucrose consumption in rodents has also been observed when oxytocin is directly injected into the VTA or ACB core, further supporting oxytocin’s role in reward processing [7,10]. Indeed, a recent study from our lab has shown that lateral ICV injection of oxytocin in rats inhibits VTA dopamine neuron activity and preferentially decreases sucrose motivation and consumption over chow consumption in a choice task [69].

Oxytocin has been extensively shown to reduce food intake and body weight in animals maintained on a high fat diet [6,13,21,142,143]; however, many of these palatable high fat diets also contain simple sugars as the primary carbohydrate source. For example, one study investigated the effect of chronic oxytocin infusions into the hindbrain (fourth ventricle) for 27 days and reported that oxytocin attenuates body weight gain, adipose mass, and reduced consumption of a high fat/high sugar diet [13]. In a similar design, Blevins and colleagues showed that chronic third ventricle oxytocin infusion into the central nervous system (CNS) attenuates weight gain, reduces food intake, and enhances sensitivity to the meal size attenuating effects of CCK [6]. Interestingly, these outcomes were observed regardless of whether sugar was included in the high fat diet or not. However, in both studies, chronic oxytocin infusions into either the third or fourth ventricle reduced food intake in high fat diet fed animals, but the same did not occur in chow-fed rats [6,13]. These findings are contrary to those of Olszewski and colleagues, who investigated the acute anorexigenic effect of oxytocin in rodents on high fat foods and revealed that oxytocin antagonist injection did not affect intralipid intake but increased sucrose intake in mice [138].

Some of the earliest observations that oxytocin reduces food intake were performed in rats fed standard chow [4,5]. Notably, most standard rodent chow diets contain a high percentage of calories from carbohydrates and, therefore, it is possible that the intake reducing effects of chow are dependent on the carbohydrate composition of the chow. Oxytocin’s capacity to acutely reduce chow intake has been demonstrated when administered either to the cerebral ventricles, peripherally, or to various regions of the brain [4,21,43,144]. For example, oxytocin injection in the BLA and CEA attenuates chow intake in rats deprived of food overnight [8]. Similarly, direct oxytocin injection into either the VMH or the ARH reduces chow consumption in food-deprived rats [53,58]. Thus, taken together, acute oxytocin administration consistently reduces high carbohydrate foods (both palatable sweet solutions and bland rat chow) when administered to the aforementioned brain regions, whereas reduction of foods high in fat but not sucrose may require chronic long-term administration in cerebral ventricles.

### 5.2. Satiation/Meal Size Control

As also discussed above in Section 2.1, oxytocin reduces food intake in part by augmenting the satiating capacity of various physiological meal termination signals. These effects are likely mediated, in part, via a descending hindbrain pathway as PVH oxytocin neurons—primarily those located in the caudal part of the parvocellular division—project to the dorsal vagal complex (DVC) [41,145]. Meal-related signals induced by preload ingestion elevate DVC oxytocin content, and NTS OT-R signaling enhances the intake inhibitory effects of various endogenous GI satiation signals [12,38]. Hindbrain oxytocin signaling enhances the satiating effect of CCK and leptin, and administration of an oxytocin antagonist can even blunt leptin’s ability to enhance CCK activation of the NTS [37]. Together, these findings demonstrate that oxytocin plays a part in endogenous satiation signaling by various categories of within-meal physiological signals.

### 5.3. Reward

Oxytocin plays a role in reducing rewarding aspects of food intake and learned food-motivated behaviors. For example, oxytocin neurons project to various regions in the brain reward circuitry, including VTA and SNc, ACB, PFC, and extended amygdala [65,70,72,74,146]. Emerging evidence suggests that oxytocin acts directly within the mesolimbic pathway (ACB core, VTA) to inhibit food intake [7,10,47]. Further, oxytocin delivered to the cerebral ventricles, NTS, or VTA reduces motivation to work for palatable food and reduces re-instatement of food-seeking behavior [47]. As briefly mentioned above, we recently reported [69] that oxytocin reduces palatable food-seeking behavior in a conditioned place preference task, impulsive operant responding for palatable food, and motivation to work for sucrose in a free chow vs. operant sucrose choice task. These outcomes are likely mediated, in part, via action in the mesolimbic dopamine pathway as we demonstrated that ICV oxytocin reduces dopamine neuron activity in response to Pavlovian cues associated with sucrose access. However, central oxytocin does not affect incentive learning in a procedure where sucrose motivation is increased when sucrose is consumed following an energetic motivational shift (from satiety to hunger/fasted) [69]. These data are consistent with a previous study demonstrating that instrumental incentive learning is not affected by treatment with flupenthixol, a D1 and D2 receptor antagonist [147], thereby suggesting that instrumental incentive learning is independent of dopamine signaling. Another recent study found that oxytocin bath application (ex vivo brain slices) or optogenetic stimulation of oxytocin neurons decreases excitatory synaptic transmission in VTA dopamine neurons via long lasting, presynaptic, endocannabinoid-dependent mechanisms [66]. Thus, together these findings suggest a possible neural mechanism through which oxytocin may decrease mesolimbic dopaminergic signaling to reduce palatable food-seeking behaviors.

### 5.4. Social

Oxytocin is well-studied in the context of social behavior, and regulates various aspects of social recognition and discrimination, memory, bonding, reproduction, and parental care [148]. Oxytocin’s importance in the regulation of social function appears to interact with its role in eating behavior. For example, despite the well characterized anorexigenic effect of central oxytocin in a singly housed setting, oxytocin does not always decrease food consumption occurring in a social context [149]. Specifically, while activation of OT-Rs in the ACB core decreases palatable food tastants in an isolated/non-social setting, ACB oxytocin is ineffective at reducing intake during a social meal [7]. Oxytocin effects on food intake are also sensitive to within-group relationships for socially housed mice. In dominant mice, for example, administration of an OT-R antagonist increases sugar intake regardless of social context, but in subordinate animals it is only effective in a non-social context [150]. Together these results suggest that oxytocin effects on food intake are variable based on social relationships. These effects may result in part from oxytocin’s interaction with the dopaminergic system to modulate attention-orienting responses to external social cues, where oxytocin may enhance dopamine’s effect on salience coding [151]. These responses are necessary for social characterization of others into in- or out-group, resulting in favoritism and co-operation of members of the in-group, as well as defense and competition with members of the out-group [152,153]. Future studies are necessary to examine the dynamic relationship between oxytocin and social feeding behaviors, and to what extent the effects of oxytocin on dopamine signaling are reinforcement specific.

### 5.5. Competing Motivations

In addition to food intake and social behavior, oxytocin impact several other behaviors, including reproduction, fear, and stress [154]. It has been suggested that these behaviors may be in competition, and thus oxytocin may reduce eating in part to divert attention and/or resources to another behavioral outcome [155]. This section reviews the impact of oxytocin on these competing motivations as it relates to the impact of oxytocin on food intake control.

Recent findings from our lab demonstrate that sex and estrous stage interact with oxytocin to affect feeding behavior. Specifically, central oxytocin administration is less effective at reducing chow intake in randomly cycling female rats in comparison to males. Furthermore, estrous stage and estrogen administration in OVX female rats enhances oxytocin’s anorexigenic effects [9]. Given that food intake and mating are mutually exclusive behaviors, it is possible that oxytocin interacts with estrogen to further inhibit food intake and focus on mating behaviors, at least in females. The oxytocin system also changes in response to pregnancy and its response to many stimuli (such as stress) are attenuated [156] through progesterone/opioid dependent mechanisms [157]. During pregnancy, body weight increases to prepare for the metabolically demanding act of birth/lactation [158,159]. Starting in mid-pregnancy, the excitability of oxytocin neurons is reduced, and central dendritic oxytocin release is inhibited contributing to maternal hyperphagia [158]. Additionally, oxytocin receptor binding patterns change [158]. While oxytocin responsiveness to CCK is enhanced, opioid inhibition restrains the response [160,161]. These processes prevent loss of accumulating oxytocin stores needed for birth [160], and lead to increased food intake [158]. Prolactin, which is necessary for milk synthesis, also influences oxytocin activity during lactation. While prolactin inhibits oxytocin neurons in virgin and pregnant rats, prolactin activates oxytocin neurons in lactating rats, thus allowing for concurrent activation for milk synthesis and delivery [162]. Moreover, prolactin-releasing peptide activates oxytocin neurons in response to food intake or CCK administration, thereby contributing to meal termination [14]. Overall, these findings suggest that oxytocin’s influence on food intake changes throughout mating, pregnancy, and lactation based on the different energetic demands during these stages.

In males, oxytocin may reduce food intake, in part, by stimulating sexual arousal. For example, oxytocin released from axonal varicosities originating from the PVH promotes penile erection in males [97]. Interestingly, Caquineau and colleagues found that acutely food-restricted male rats maintain their sexual motivation towards receptive females; however, mating initiation is delayed when these males are placed in cages with receptive females [163]. The delay corresponded with reduced c-Fos expression in oxytocin neurons in the lateral posterior parvocellular region of the PVH compared with levels of fed rats paired with receptive females. The authors concluded that the desire for food competes with the motivation to mate when in a hungry state; that reproductive behavior can be altered by nutrition status possibly via oxytocin signaling in the brain [163].

The effect of oxytocin on eating with respect to competing motivations with social behavior has also been investigated [150]. For example, as mentioned above in Section 5.4 Olszewski et al. observed that when subordinate mice injected with an IP oxytocin antagonist were exposed to their dominant counterparts partially and/or fully in the same context, the subordinate group’s sucrose solution intake was reduced [150]. However, in the absence of any social cues related to the dominant animal, the antagonist injection increased sucrose intake in subordinate mice. Conversely, in dominant mice, oxytocin receptor antagonism increased sucrose intake regardless of whether the animals were in a social setting. Moreover, oxytocin mRNA expression in the hypothalamus between dominant and subordinate mice varied, with the dominant group showing higher oxytocin mRNA expression in the full social environment when compared to the subordinate group [150]. These findings imply that social context can impact efficacy of the oxytocinergic system to reduce food intake.

Collectively, familiarity, social hierarchy, and reproduction-related social and other factors appear to influence oxytocin’s effect on feeding. Future studies are necessary to further examine oxytocin’s effect on other types of social eating behaviors, such as with non-familiar vs. familiar conspecifics.

## 6. Concluding Framework

The literature reviewed above is consistent with the framework that oxytocin’s inhibitory effects on food intake are mediated by enhancing within-meal satiation signaling to reduce meal size rather than by modulating between-meal interoceptive hunger or satiety states. More specifically, the collective literature indicates that a primary mechanism through which oxytocin reduces food intake is to boost satiation signals to terminate an ongoing meal via hindbrain OT-R signaling. The distributed OT-R signaling across the neuroaxis likely involves a combination of wired synaptic signaling (from oxytocin neuron projections), as well as non-synaptic volume transmission through the interstitial and/or cerebrospinal fluid in the brain.

Several studies reviewed herein suggest that oxytocin reduces food intake in a macronutrient-dependent manner, preferentially decreasing the intake of palatable, sweet foods. Consistent with these findings, oxytocin also plays a role in reducing rewarding aspects of eating and learned food-motivated behaviors. In addition to food intake regulation, oxytocin impacts several other behaviors, which may be in competition with the drive to eat, and thus oxytocin may reduce eating, at least in part, to divert attention and/or resources to another competing behavioral outcome. This possibility represents an important area for future investigations into this system, as does investigating oxytocin volume transmission signaling and OT-R-mediated effects on food intake in telencephalic brain regions that regulate cognitive processes.

## Data Availability

Not applicable.
